# Kikuchi-Fujimoto Disease: A Rare Cause of Cervical Lymphadenopathy

**DOI:** 10.7759/cureus.17021

**Published:** 2021-08-09

**Authors:** Zubayer Ahmed, Huma Quadir, Knkush Hakobyan, Mrunanjali Gaddam, Amudhan Kannan, Ugochi Ojinnaka, Jihan A Mostafa

**Affiliations:** 1 Internal Medicine, California Institute of Behavioral Neurosciences & Psychology, Fairfield, USA; 2 Internal Medicine/Family Medicine, California Institute of Behavioral Neurosciences & Psychology, Fairfield, USA; 3 Diagnostic Radiology, California Institute of Behavioral Neurosciences & Psychology, Fairfield, USA; 4 General Surgery, California Institute of Behavioral Neurosciences & Psychology, Fairfield, USA; 5 Family Medicine, California Institute of Behavioral Neurosciences & Psychology, Fairfield, USA; 6 Psychiatry, California Institute of Behavioral Neurosciences & Psychology, Fairfield, USA

**Keywords:** kikuchi-fujimoto disease, lymphadenopathy, autoimmune diseases, immunohistochemistry, viral infection

## Abstract

Kikuchi-Fujimoto disease (KFD) is a rare benign disease, clinically characterized by fever and tender cervical lymphadenopathy affecting the posterior cervical lymph nodes. This disease is usually accompanied by night sweats, rashes, and headaches. It generally affects young individuals, especially females, of Oriental-Asian origin. The etiology of KFD remains uncertain, but associations have been noted with viral diseases including Epstein-Barr virus (EBV), herpes simplex virus (HSV), and varicella-zoster virus (VZV), as well as autoimmune disorders including systemic lupus erythematosus (SLE) and Sjogren's syndrome. This review points out the etiology of KFD with cervical lymphadenopathy alongside its clinical presentation, histological highlights, lab investigations, complications, and treatment. Accurate diagnosis of this disease depends on lymph node excisional biopsy. Three histological patterns of KFD are recognized: proliferative, necrotizing, and xanthomatous. Distinction from lymphadenopathy-associated alternate disorders (e.g., SLE, malignancy, tuberculosis, or another infectious lymphadenitis) is essential to ensure appropriate therapy. This self-limited condition entails nonsteroidal anti-inflammatory drugs (NSAIDs) for pain relief with consideration of corticosteroids and hydroxychloroquine in severe cases.

## Introduction and background

The Kikuchi-Fujimoto disease (KFD) was first described in 1972 by Japanese scientist Seishi Kikuchi, who identified it as a rare benign disorder, and by Y. Fujimoto, who reported it separately in the same year. The disease commonly causes mild fever, tender cervical adenopathy, night sweats, and headache. Weight loss, skin rash, nausea, vomiting, and sore throat can also be associate with KFD [1, 2]. It resembles other diseases associated with lymph nodes. Previous reports have shown that KFD commonly affects young adults of Asian origin, more frequently females. However, research from several Asian countries indicated that both males and females are equally affected.

In clinical research, there is no relation found between KFD and viral infection. However, studies show that KFD causes T-cells to induce an immunological response to diverse antigens in genetically predisposed individuals. Patients with KFD have specific human leukocyte antigen (HLA) class II alleles, notably HLA-DPA1 and HLA-DPB1. Asians more commonly carry these alleles than non-Asians, who are extremely rare carriers [3, 4].

According to several physicians, KFD may be associated with autoimmune diseases such as systemic lupus erythematosus (SLE), Sjogren's syndrome. Polymyositis, scleroderma, lymphoma, and thyroiditis are also associated with KFD. Patients have experienced changes similar to those suffering from SLE and other autoimmune diseases [3]. The disease is diagnosed by lymph node excision biopsy. A self-limiting disorder, the symptoms of KFD usually resolve within a few weeks to months [4, 5].

This review article aims to find out the particular etiology, clinical presentations, histological features, lab investigations, complications, and treatment of KFD. In addition, we briefly discussed recent information about KFD and reviewed the relationship between KFD and autoimmune diseases like SLE.

## Review

Epidemiology

The disease usually affects people aged below 30; however, KFD can also affect other age groups, including children. Some studies show female preponderance (4:1) in KFD [5]. However, new investigations have reported equal distribution of the disease among the male and female Asian populations [2]. Additionally, some rare case studies in the United States (US) and Europe found that the disease has been distributed in all racial groups and ethnic groups [6]. Human leukocyte antigen (HLA) genotype studies showed a connection between KFD and HLA-DPB1 and HLA-DPA1 alleles in the Asian population. Genetic testing is not usually done in clinical settings [7]. There is unclear relation of KFD to environmental factors [8, 9].

Clinical presentations

The onset of KFD could be intense or sub-acute and persist chronically in a sporadic condition. Symptoms can last up to weeks and, in some cases, even months. Unilateral or bilateral cervical tender lymphadenopathy associated with low-grade fever typically occurs in the posterior cervical triangle. The presence of KFD on the anterior cervical lymph nodes is not always the case. The size of cervical lymph nodes has been reported to vary from 0.5cm to 4cm [4]. However, lymph node sizes can range from 5cm to 6cm and are rarely larger than 6cm. The disease can cause generalized lymphadenopathy, though it is pretty rare. A low-grade fever may be reported in 50% of KFD patients, along with respiratory symptoms such as cough and headache. Less frequent symptoms of KFD include skin rash, weight loss, nausea, vomiting, sore throat, and night sweats [1, 2, 10]. Apart from fever and tender cervical lymphadenopathy, leukopenia has been detected in 50% of KFD patients. Atypical lymphocytes, similar to those identified in EBV infection, are seen in peripheral blood studies. Extranodal involvement is relatively infrequent in KFD. However, this condition has been linked to skin, eye, and bone marrow infection, as well as liver malfunction [4].

Splenomegaly and hepatomegaly can also occur in 5% of cases. The bone marrow and nervous system involvement have also been reported among a few KFD patients [4, 11]. About 40% of KFD patients are likely to experience skin issues that might manifest as a nonspecific rash. The disease may further cause erythema multiforme, which can be maculopapular, morbilliform, nodular/popular, ulcer on the mucous membrane, malar erythema, alopecia, and lupus-like skin lesions [11, 12]. Purpura or petechial rashes have also been observed in acute presentations. A biopsy of the skin lesion might further reveal the presence of vacuities [13, 14]. Clinically, as compared to adults, children are more prone to severe and protracted fevers and have a higher risk of lymph node necrosis [15].

The disease is often linked to an autoimmune or viral etiology, resulting in an overactive T-cell-mediated immune response [5]. It is sometimes related to *Yersinia enterocolitica* and *Toxoplasma Gondi* infections. However, subsequent studies failed to validate their relationships because of variable types of lymphadenitis features [5]. Additionally, Epstein-Barr virus (EBV) and cytomegalovirus (CMV) may be considered in the pathogenesis of KFD. Other differentials of KFD include cat scratch disease and AIDS [6].

In a case study of a 24-year-old Nepali female, the patient presented to the hospital's outdoor service with fever, headache, and tender and distinct left cervical and axillary lymph nodes. These symptoms were prevalent for approximately three weeks. Tests revealed a high erythrocyte sedimentation rate (ESR) and thrombocytopenia. Physical examination showed tender lymphadenopathy and persistent low-grade fever, indicating bacterial lymphadenitis and tuberculosis. An excised cervical lymph node was sent for histopathological examination to achieve a definitive diagnosis. The diagnosis was KFD, based on the pathognomonic features of paracortical foci composed of various types of histiocytes, including crescentic type, in the background of abundant apoptotic karyorrhectic debris. In a follow-up visit after six weeks, the patient had no significant systemic symptoms; the enlarged and swollen lymph nodes had significantly decreased in size, ESR and platelet count had become normal [16].

Low-grade fever and localized lymphadenopathy of KFD might be mistaken for tuberculosis [16]. Consequently, it serves as the most prevalent preliminary diagnosis for any long-term non-tender lymphadenopathy. Both illnesses also cause nocturnal sweats and weight loss. Some disorders are also considered as the differential diagnosis of KFD. Similar clinical manifestations to KFD include SLE, infectious mononucleosis,non-Hodgkins lymphoma toxoplasmosis, and cat-scratch disease. Fine-needle aspiration cytology (FNAC) is less invasive and cost-effective, and the diagnostic time is quicker; however, its accuracy is only 56%. If unequivocal cytological characteristics are observed, FNAC can be used to diagnose KFD with clear-cut clinical symptoms. However, upon observing atypical clinical symptoms, it is recommended to perform a histological assessment of the affected lymph nodes for diagnosis [17, 18].

The disease can sometimes be associated with malignancies, as both illnesses are linked to fever and cervical adenopathy [7]. In addition, some infections of the central nervous system, renal failure, parenchymal, and pleural lung disease are all linked to KFD. This suggests that certain physical traumas and malignancies can trigger the onset of the disease. Based on the foregoing discussion, a viral or unknown infectious pathogen triggers an inflammatory response and causes KFD.

Diagnostic investigations

Histopathological Findings

Based on the results in histology and immunohistochemistry, it is seen that an overwhelming reaction to numerous organisms may cause KFD lymphadenopathy with fever [2]. In addition, genetically predisposed individuals have an immoderate T-cell-mediated immune response to different nonspecific stimuli.

A KFD patient's lymph nodes histologically show follicular hyperplasia with the partially preserved architecture of the lymph node. The paracortex area looks patchy, expanded. Karyorrhexis cells and a significant accumulation of histiocytes are found at the edge of necrosis. Isolated apoptotic cells scattered throughout large sheets of histiocytes, admixed with cellular debris and nuclear dust are sometimes found. In the necrotic foci, there are frequent so-called crescentic histiocytes. Small lymphocytes, activated T cells, and some plasma cells are seen among the histiocytes. Neutrophils and eosinophils are absent [7, 9]. At the edge of necrotic areas, plasmacytoid dendritic cells, as well as a lymphocyte, are seen. In the periphery of necrosis, area clotted blood vessels are found.

The most common histologic findings of KFD patients are the proliferative, necrotizing, and xanthomatous patterns. An expanded paracortex with sheets of histiocytes and plasmacytoid dendritic cells admixed with small lymphocytes and karyorrhectic nuclear debris is found in the initial proliferative pattern. A necrotic phase of the histology is characterized by the presence of necrotic tissue on the lymph nodes. An abundance of foamy histiocytes, with or without necrosis found in the xanthomatous phase [3, 14].

Apart from cervical lymph nodes, mediastinal and axillary lymph nodes are also involved in KFD. Sometimes generalized lymphadenopathy is also found. Histology of skin biopsy of KFD patients has shown atrophy of the epidermis, basal cell vacuolation, focal hyperkeratosis, pilosebaceous gland atrophy, and follicular plugging. In addition, edema and a lymphocytic infiltrate in the upper dermis are also seen. These features suggest SLE but can also be found in KFD patient skin findings [8].

 Lab Findings

No accurate diagnostic laboratory tests or procedures have yet been developed to diagnose KFD with fever and cervical lymphadenopathy [7]. Elevated lactate dehydrogenase, low neutrophil count, lymphocytosis, and leukopenia are found on KFD patients [15]. Also, abnormal liver enzymes and elevated ESR are seen in laboratory investigations. There are abnormally elevated liver enzymes due to the involvement of the liver, spleen, and lymph nodes. The diagnosis of KFD thus primarily depends on the lymph node excisional biopsy in typical clinical symptoms.

However, serologic testing for antibodies against these infections has consistently shown negative results, and no viral particles have been found [5]. In addition, the Polymerase Chain Reaction (PCR) test failed to reveal a positive association between KFD and the four common suspected viral agents EBV, HHV-6, HHV8, and parvovirus B19. Furthermore, serologic testing of antinuclear antibodies (ANA) has been found consistently negative for KFD [5].

 *Imaging Findings*

The preferred method to examine cervical lymph nodes is USG. In the case of children, USG is the best tool to diagnose enlarged lymph nodes on the neck because it has no radiation hazard. However, the use of USG is limited in examining the internal necrosis of affected nodes. Unfortunately, only a limited number of study participants were examined using color Doppler USG. This number was relatively small to enable the analysis of the significance of imaging findings. A case study of the Korean population mentioned that the color Doppler USG findings of KFD include symmetrical central hilar vascularity with some flow signals in the peripheral soft tissue - similar to those of benign lymphadenitis. Further studies are required to use color Doppler USG in diagnosing KFD in the pediatric population [13].

It is important to note that widespread macroscopic necrosis of affected nodes was not unusual on CT scans, especially in febrile children. Thus, clinically and radiologically, KFD could be a misdiagnosis of tuberculosis or sometimes malignant lymphoma. In the CT scan of the neck, numerous homogenously enhancing nodes without nodal necrosis are observed. Additionally, in some patients, multiple 18 F-fluorodeoxyglucose (FDG)-avid nodular masses in both cervical and abdominal lymph nodes, similar to lymphoma features, were seen on FDG positron emission tomography (PET) scans. Studies examining these PET scans have indicated that the affected nodes in KFD demonstrate FDG uptake, which is compatible with malignant lymph nodes. The most common CT feature of KFD in children was the clusters of multiple, small to medium-sized ovoid and round lymph nodes associated with internal necrosis [19].

Prognosis and complications

The disease usually resolves within months without any specific treatment. The relapse rate of KFD is about 3-4% in adults. In chronic cases, treatment involves corticosteroids, intravenous immunoglobulin, or hydroxychloroquine [11, 14]. A study found that removing the affected lymph node has both a diagnostic and therapeutic effect. Although the diagnosis of KFD is climacteric, the overall prognosis of KFD is satisfactory. Some studies have also shown that KFD can progress to SLE after a few months or years of diagnosis. After the diagnosis, KFD patients must do routine follow-up with the primary doctor for any complications.

Hemophagocytic lymphohistiocytosis (HLH), a relatively rare condition, can be found in KFD patients. In this condition, a robust immunologic response is associated with histolytic proliferation, hemophagocytosis, and systemic inflammatory response. In addition, disseminated intravascular coagulation can also occur in hemophagocytic lymphohistiocytosis that can be life-threatening. The mortality rate of HLH is 20-42%. It is managed with intravenous immunoglobulin and methylprednisolone. Pediatric patient prognoses are typically better than that of adults.

The disease is self-limiting but neurologic complications such as meningoencephalitis, cerebellar ataxia, and encephalitis with CNS lesions can occur in rare cases. Aseptic meningitis is also seen to be associated with KFD [16].

Treatment

Effective treatment for KFD is still challenging. Symptomatic KFD patients are usually treated with anti-inflammatory therapies such as NSAIDs [3]. In severe conditions of KFD, corticosteroids can be used. Studies show that hydroxychloroquine, alone or in combination with glucocorticoids, benefited symptomatic KFD patients. A hypothesis has also been proposed that combining anti-inflammatory medication and immunosuppressive treatment to the treatment regimen becomes a drug of choice for symptomatic patients [17].

**Figure 1:**
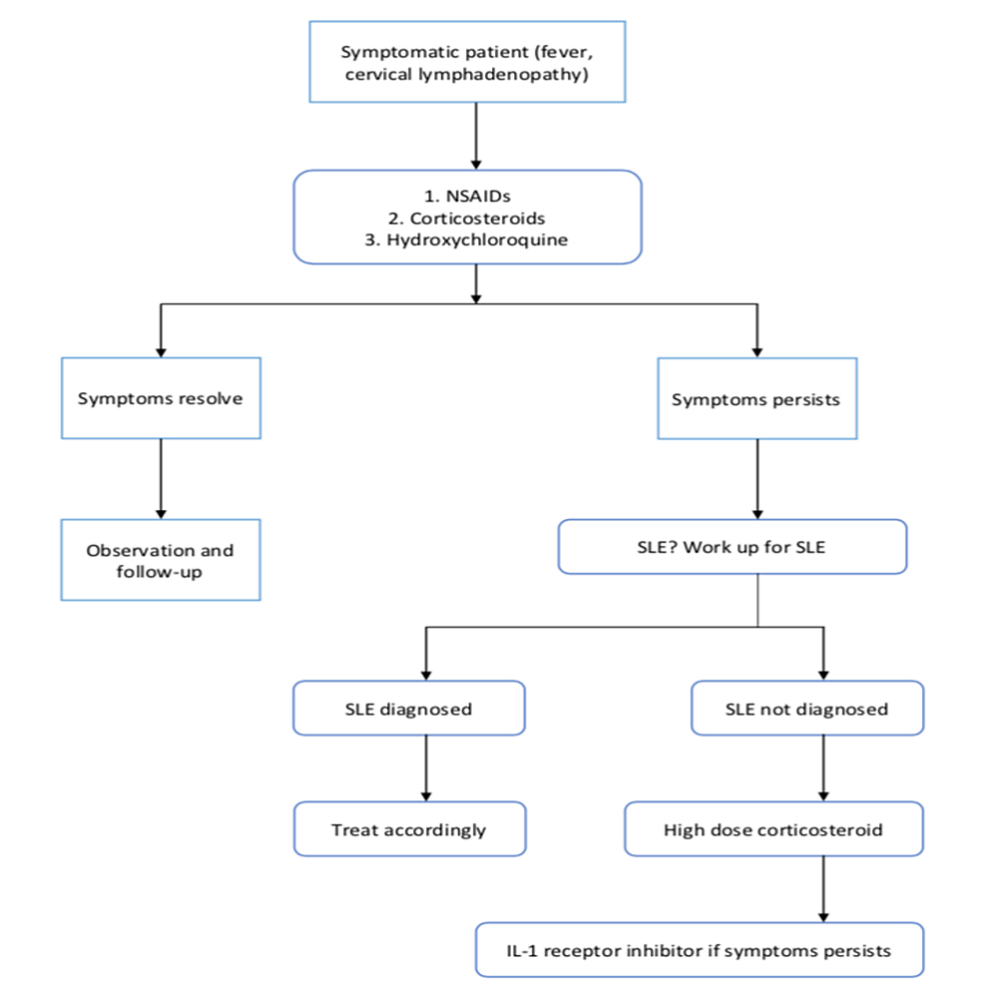
Flow chart showing the treatment of Kikuchi-Fujimoto disease (KFD) NSAIDs: Nonsteroidal anti-inflammatory drugs; SLE: Systemic lupus erythematosus; IL-1: Interleukin-1

This disease can also be associated with SLE, Sjogren's syndrome, and viral diseases such as EBV. The patients must be reassured that KFD is a self-limiting condition, and the patient's family members should be informed about the peaceful state of the disease. The treatment flow chart of KFD is shown in Figure 1.

Corticosteroids showed great benefit but only initially. If any patient has multiple recurrences of KFD, we can use anakinra, which is a recombinant human interleukin-1 (IL-1) receptor inhibitor. It blocks the downstream inflammatory actions of IL-1 alpha and IL-1beta. The human IL-1 inhibitors have a strong steroid-sparing effect. Therefore, we can use anakinra in case of treatment resistance or recurrent cases of KFD [20]. Unfortunately, KFD has a recurrence rate of 3% [21].

Limitation

In this traditional review, we have included only a few studies, and some studies have a small number of samples. With this restricted number of studies and data, we tried to discuss recent information about KFD, its etiology, variable clinical presentation, histological features, lab investigations, and treatment options. Another limitation of this traditional review is that KFD is a rare infectious disease. 

## Conclusions

The rarity and variable histologic and clinical presentation of KFD creates significant diagnostic and therapeutic challenges to clinicians. Lymph node biopsy is best for diagnosing KFD. Patients should be reassured that it is a self-limiting condition, and family members should be comforted. Given the variable clinical presentation and impressive rarity of KFD, studies should continue to find out more information about this disease. Research should continue until the primary etiology of the disease is found. Many clinical studies showed that KFD could occur due to an immune response to underlying autoimmune diseases, viral diseases, or bacterial infections like tuberculosis. Therefore, the diseases associated with KFD also need to be studied to determine the relationship between these diseases. An interprofessional team that includes internists, pathologists, rheumatologists, and infectious disease experts best manages the disease. Information sharing between health professionals will help determine the etiology, prognosis, pathophysiology, and definitive treatment over the long term.
